# Associations between maternal diet quality in pregnancy and infant feeding practices

**DOI:** 10.1017/S000711452500025X

**Published:** 2025-03-14

**Authors:** Meaghan J. Sexton-Dhamu, Katherine M. Livingstone, Ewa A. Szymlek-Gay, Li Ming Wen, Miaobing Zheng

**Affiliations:** 1 Institute for Physical Activity and Nutrition, School of Exercise and Nutrition Sciences, Deakin University, Geelong, Australia; 2 School of Public Health and Sydney Medical School, The University of Sydney, Sydney, Australia; 3 Health Promotion Unit, Population Health Research and Evaluation Hub, Sydney Local Health District, Sydney, NSW, Australia

**Keywords:** Diet quality, Maternal, Pregnancy, Breast-feeding, Complementary feeding

## Abstract

Postpartum maternal diet quality has been linked with optimal infant feeding practices. However, whether maternal diet quality during pregnancy influences infant feeding practices remains unclear. The present study explored the relationship between maternal diet quality in pregnancy and infant feeding practices in Australian women. A brief 15-item FFQ was used to collect maternal dietary data (*n* 469). Diet quality was calculated using a modified 2013 Dietary Guideline Index (DGI). Multivariable linear and logistic regressions with adjustment for covariates were used to examine associations between maternal diet quality in pregnancy and infant feeding practices: infant feeding mode, breast-feeding duration and timing of solids introduction. Higher DGI score during pregnancy was associated with higher odds of breast-feeding than formula/mixed feeding (adjusted OR (AOR) 1·03, 95 % CI 1·00, 1·07), longer breast-feeding duration (adjusted *β* 0·09, 95 % CI 0·03, 0·15) and higher odds of breast-feeding for ≥ 6 months (AOR 1·04, 95 % CI 1·02, 1·07) than for < 6 months. Associations between maternal DGI score and breast-feeding variables were moderated by maternal country of birth, with significant associations observed in Australian-born mothers only. No association was found between maternal DGI score and timing of solids introduction. Higher maternal diet quality was associated with better infant feeding practices, and the association was moderated by country of birth. Our findings provide evidence to support the initiation of dietary interventions to promote diet quality during pregnancy, particularly among Australian-born women. Further research could explore underlying mechanisms linking maternal diet quality and infant feeding practices.

The first year of life is a critical window for the development of future dietary behaviours and long-term health outcomes^(1)^. Infant feeding practices play an important role in child health^(2)^; breast-feeding, for instance, is the foundation of infant nutrition. Longer duration of breast-feeding has many health benefits for the child, such as a lower risk of type 1 diabetes, asthma^(3)^, sudden infant death syndrome, obesity and neurological conditions^(4)^, and for the mother, such as a lower risk of breast and ovarian cancer^(5)^. Conversely, formula feeding has been linked to an increased risk of infections, asthma and atopic allergies, metabolic disease and obesity^(6)^. Some evidence also supports an association between early introduction of solids before age 4 months and adverse health consequences, including higher obesity risk^(3,7)^. The WHO and Australian infant feeding guidelines recommend exclusive breast-feeding for the first 6 months of an infant’s life^(8,9)^. Solid foods are recommended to be introduced around 6 months of age to meet the infant’s additional energy and nutrient needs^(10)^. Despite these recommendations, suboptimal infant feeding practices are highly prevalent globally^(11)^ and in Australia^(12)^. A 2010 Australian national survey of infant feeding practices reported that most women (96 %) initiated breast-feeding, but rates of exclusive breast-feeding declined sharply to 2·1 % when infants were 6 months of age^(12)^. Similarly, 35·3 % of infants were introduced to solids before 4 months of age^(12)^. In addition, 34 % of infants were introduced to formula before 1 month of age, increasing to 69 % at 6 months of age^(12)^. Therefore, it is vital to improve infant feeding practices to ensure long-term child health outcomes.

Prior research has highlighted maternal influences on child health outcomes and dietary behaviours^(13,14)^. Previous studies in various countries, including Australia^(15)^, the USA^(16)^ and Finland^(17)^, have shown that a better pregnancy or postpartum maternal diet quality was associated with better infant feeding practices, including longer breast-feeding duration and introducing solids after age 6 months. However, no studies have investigated the associations between maternal diet quality in pregnancy and infant feeding practices in Australian women.

Evidence has also revealed that maternal diet quality or infant feeding practices are influenced by maternal factors such as age, educational attainment and country of birth^(18,19)^. Therefore, the association between maternal diet quality and infant feeding practices could differ by maternal factors, but no studies have examined this to date. Thus, this study aimed to (1) investigate the associations between maternal diet quality in pregnancy and infant feeding practices (i.e. breast-feeding duration, timing of solids introduction and feeding mode) and (2) explore potential moderators. Knowledge of how maternal diet quality in pregnancy influences infant feeding practices and the potential moderators underlying this association will inform the design of dietary interventions in pregnancy and identify women at risk of poor diet quality and suboptimal infant feeding practices.

## Methods

### Study design and participants

The present study used longitudinal data from the Healthy Beginnings Trial, a randomised controlled trial to prevent early childhood obesity through a home-based intervention involving first-time mothers and their children^(20–22)^. Details of the trial and results have been published elsewhere^(20–22)^. The trial comprised a 2-year intervention stage conducted between 2007 and 2010 and a 3-year follow-up stage between 2011 and 2014. The intervention consisted of eight home visits by a trained early childhood nurse – one antenatal visit at 30–36 weeks gestation and seven postnatal visits starting from birth to 24 months^(21,23)^. During these visits, women were informed about healthy infant feeding, child and family physical activity and nutrition and social support. Nurses also provided telephone support. The control group received the usual care.

Women attending one of two antenatal clinics in metropolitan South Western Sydney were invited to participate in the trial^(20–22)^. Women were eligible to participate if they were aged 16 years or over, 24–34 weeks pregnant, were local residents and could give informed consent. Additionally, women or their guardians were required to communicate in English. Following childbirth, women were excluded from the trial if their infant was diagnosed with a medical condition influencing physical activity, eating behaviours, weight or height/length. This study was conducted according to the guidelines laid down in the Declaration of Helsinki, and all procedures involving human subjects were approved by the Sydney South West Area Health Service ethics review committee (RPAH Zone, Protocol No X10-0312 and HREC/10/RPAH/546) and the Deakin University Human Ethics Advisory Group—Health (HEAG-H 194_2021). Written informed consent was obtained from all subjects. This study is reported per the Strengthening the Reporting of Observational Studies in Epidemiology (STROBE-nut) guidelines (online Supplementary Table 1)^(24)^.

### Dietary intake

Dietary data were collected at baseline (24–34 weeks gestation) using a 15-item semi-quantitative FFQ^(25)^. The FFQ comprised ten dietary questions from the New South Wales Population Health Survey Australia^(26,27)^ along with five additional dietary questions on bread, milk and beverage intake. The FFQ captured the frequency of intake (i.e. times/serves per day, week or month or rarely/never consumed) of vegetables, fruit, bread, breakfast cereal, cooked grains/cereals, milk, processed meat, takeaway meals/snacks, potato products, sugar-sweetened beverages, fruit juice and water^(27)^. Participants also had the option of selecting ‘don’t know’ or ‘refused’. Online Supplementary Table 2 details the FFQ’s dietary questions, response options and adaptations.

### Diet quality

The 2013 Dietary Guideline Index (DGI) was used to assess maternal diet quality at baseline (online Supplementary Table 3)^(28)^. The DGI is a food-based score that assesses adherence to age- and sex-specific recommendations of the 2013 Australian Dietary Guidelines^(29)^. The DGI comprises thirteen components: seven encouraged (i.e. food variety, vegetables, fruit, grains/cereals (i.e. total cereal intake and type of bread consumed), lean meats and alternatives (i.e. total lean meats and alternatives and proportion of lean meats and alternatives to total meats and alternatives), dairy and alternatives, fluid intake (i.e. total fluid intake and proportion of water to total fluids)) and six discouraged (i.e. discretionary foods, saturated fat (i.e. trim fat from meat and type of milk consumed), unsaturated spreads and oils, salt intake (i.e. salt added during cooking and salt added during meal), added sugar and alcohol intake)^(28)^. Each component is scored 10, giving a total possible score of 130. For the present study, a modified DGI was used due to the availability of dietary data. The primary modifications were that diet variety, type of bread consumed, lean meat and alternatives intake, whether fat was trimmed from meat, salt intake and alcohol intake were excluded. Consequently, the modified DGI comprised eight components: vegetables, fruit, dairy and alternatives, fluid intake, discretionary foods, added sugar, grains/cereals (i.e. total cereal intake) and saturated fat (i.e. type of milk consumed). The sub-components grains/cereals and saturated fat were scored out of five. Scoring was categorical for saturated fat intake. Discretionary foods were reverse-scored. ‘Don’t know’ or ‘refused’ responses were assigned as missing and excluded from the analysis. Scoring was proportional for the remaining sub-components and components, with a maximum score suggesting that national recommendations had been met. The total score for the modified DGI ranged from 0 to 70, with a higher score suggesting better diet quality. For outcomes that showed a significant association with the continuous DGI score, further analyses were conducted to assess linear dose–response relationships by using DGI tertiles (i.e. tertile 1 = low, tertile 2 = moderate and tertile 3 = high), with DGI tertile 3 indicating better diet quality compared with DGI tertile 1.

### Infant feeding practices

Information on infant feeding practices was collected using a questionnaire at 6 months postpartum via telephone interview and at 12 and 24 months postpartum via face-to-face interview in participants’ homes^(20,22)^. Detailed information on the questionnaire has been previously published^(30)^. At 6 and 12 months postpartum, women were asked whether their child had ever been breastfed, the total time their infant had been breastfed in weeks and months and at what age (in weeks and months) their child had been given infant formula or solid food regularly. At 24 months postpartum, women reported whether they were still breast-feeding their child, had never breastfed or the age in weeks and months that their child had stopped breast-feeding. Women responded to a similar question on infant formula.

Infant feeding variables were derived by combining the infant feeding questions across three time points. They comprised of feeding mode at age 12 months, any breast-feeding duration and timing of solids introduction, which were derived by combining the infant feeding questions across three time points. Feeding mode at age 12 months was categorised as breast-feeding only or mixed feeding/formula feeding only, which was combined due to the small proportion of formula-feeding-only infants (3·8 %). Women who had never given infant formula and had breastfed for 12 months or more were grouped into the breast-feeding category. Women who had reported only ever formula feeding or who had reported both breast-feeding and formula feeding were grouped into the mixed feeding/formula feeding category. The age in weeks and months that solid food was first given was used to derive the timing of solids introduction variable. Timing of solids introduction was categorised as < 6 *v*. ≥ 6 months. Similarly, age in weeks and months that breast-feeding continued was used to calculate the breast-feeding duration variable. Two variables on breast-feeding duration were used: a continuous variable and a binary variable categorised as < 6 *v*. ≥ 6 months. Consistent with prior analyses from the Healthy Beginnings Trial^(31)^, the timing of solids introduction and breast-feeding duration variables were analysed using 6 months as the cut-off to facilitate results interpretation, according to the recommendations of the WHO^(2)^ and the National Health and Medical Research Council^(8)^ regarding exclusive breast-feeding and introduction of solids recommendations at 6 months.

### Covariates and potential moderators

Covariates and potential moderators were chosen based on a directed acyclic graph (online Supplementary Figure 1), which was constructed based on theory and the findings of prior literature^(18,32)^. Socio-demographic data of women were collected at baseline, including age (in years), pre-pregnancy BMI (kg/m^2^), country of birth (Australia *v*. other), educational attainment (high school or less *v*. trade certificate/diploma *v*. university degree or higher), employment status (employed *v*. unemployed), marital status (married/ *v*. not married) and smoking status (non-smoker *v*. past/current smoker). Child sex was categorised as boy or girl.

### Statistical analysis

Participant characteristics were summarised using descriptive analyses. Categorical and continuous variables are presented as percentages and means (SD) or medians (IQR), respectively. Analyses were conducted to assess differences in maternal diet quality scores and infant feeding variables by intervention allocation (intervention *v*. control group). No difference was found for all variables (DGI score: mean difference –0·01, 95 % CI –1·74, 1·72, *P* = 0·99; breast-feeding duration (months): mean difference –0·88, 95 % CI –2·07, 0·31, *P* = 0·15; breast-feeding duration (≥ 6 v. < 6 months): *P* = 0·18; timing of solids introduction: *P* = 0·18; feeding mode: *P* = 0·1). Consequently, the intervention and control groups were pooled for the present study, with intervention allocation included as a covariate to account for potential differences between groups. Differences between women included or excluded from the analysis were investigated using the *t* test for continuous variables and the χ^2^ test for categorical variables. Differences between DGI tertiles were explored using one-way analysis of variance for continuous variables and the χ² test for categorical variables.

Multivariable linear or logistic regressions were performed to investigate associations between maternal diet quality in pregnancy (exposure) and continuous (breast-feeding duration in months) or categorical (breast-feeding duration: < 6 *v*. ≥ 6 months; timing of solids introduction: before or at 6 months; feeding mode: breast-feeding or mixed feeding/formula feeding) infant feeding outcomes, respectively. Models were run first with maternal diet quality score as a continuous variable and then with diet quality categorised into tertiles. Crude models were adjusted for intervention allocation, and adjusted models included all covariates (i.e. maternal age, country of birth, educational attainment, employment status, marital status, smoking status, prepregnancy BMI and child sex) as well as intervention allocation. Variance inflation factors were checked for potential multicollinearity, and no significant correlations were found. *P*-trends for the association between categorical DGI tertiles and infant feeding variables were calculated using linear regression models with tertiles of diet quality analysed as a continuous variable.

Variables (i.e. maternal age, country of birth, educational attainment, employment status, marital status, smoking status, child sex, prepregnancy BMI and intervention allocation) that moderate the association between maternal diet quality in pregnancy and infant feeding practices were explored, informed by previous research^(33)^. Potential interactions between maternal diet quality and the covariates were investigated by including interaction terms in the adjusted model. Stratified analyses were conducted when significant interactions (*P* < 0·05) were observed. For models with breast-feeding duration (in months and < 6 *v*. ≥ 6 months) as the outcome, a significant interaction between maternal diet quality and country of birth was observed. Consequently, stratified analyses were performed to further test whether the associations between maternal diet quality and breast-feeding duration differed by country of birth. The likelihood ratio test revealed that the addition of an interaction term between maternal DGI and maternal country of birth significantly improved the model fit.

### Sensitivity analyses

Multiple imputations by chained equations with ten datasets were performed, imputing missing covariates (online Supplementary Figure 1), to determine their effect on the analysis. Logistic regressions were conducted to predict the missing covariates. Stata’s ‘mi estimate’ command was used to pool estimates from the ten datasets. All analyses were performed using Stata version 18·0 (Stata Corp). Results were considered significant at *P* < 0·05.

## Results

Of the 667 women recruited into the Healthy Beginnings Trial, 164 (24·6 %) were excluded for missing data on infant feeding practices, and a further 34 (5·1 %) for missing data on covariates (online Supplementary Figure 1). Thus, a total of 469 women were included in the final analysis. Compared with the included sample, more women from the excluded sample were younger, unemployed, unmarried did not go to university or had a lower DGI score in pregnancy (online Supplementary Table 4).

### Participant characteristics

Table 1 summarises the characteristics of the 469 women by DGI score and DGI tertile. Overall, women had a mean DGI score of 47·5 (sd 9·5) and a mean prepregnancy BMI of 25·0 (sd 5·6). Most women were born in Australia (*n* 305, 65·0 %), employed (*n* 280, 59·7 %), married (*n* 423, 90·2 %), a non-smoker (*n* 309, 65·9 %), had attained a trade certificate or diploma (*n* 177, 37·7 %) or had given birth to an equal number of boys and girls. Women, on average, breastfed their infants for 6·1 (IQR 0·6–9·8) months. Most women breastfed their infants for < 6 months, introduced solids at or after 6 months or used formula or mixed infant feeding modes. There were significant differences across all three DGI tertiles for maternal age, country of birth, educational attainment, employment status, marital status and smoking status (*P* < 0·05). Across all three tertiles, women were predominantly born in Australia, but the proportion of Australian-born women decreased in DGI tertiles 2 and 3. Most women in DGI tertile 1 had finished high school or less, while women in DGI tertile 3 had completed university. From DGI tertiles 1 to 3, there were increasing trends in the proportion of women who were employed or married. Most women were non-smokers across all three tertiles; however, DGI tertile 1 had the smallest proportion of non-smokers compared with the other two tertiles. There was a non-significant difference (*P* = 0·4) in child sex across the DGI tertiles. There were significant differences across all DGI tertiles for breast-feeding duration (in months and > 6 *v*. ≥ 6 months) and feeding mode (*P* < 0·05). Compared with women in DGI tertiles 2 and 3, women in DGI tertile 1 breastfed their infants for a shorter duration and predominantly used formula or mixed feeding practices. Between tertile 2 and tertile 3, breast-feeding duration decreased slightly. There was a non-significant difference (*P* = 0·07) in the timing of solids introduction across tertiles.

**Table 1.** Demographic characteristics of women from the healthy beginnings trial (*n* 469) (Numbers and percentages; mean values and standard deviations; median values and interquartile ranges)

	Overall		DGI tertile 1		DGI tertile 2		DGI tertile 3	
Characteristic	*n*	%	*n*	%	*n*	%	*n*	%
Maternal characteristic								
Age at baseline, years								
Mean	27·1		25·5		27·0		28·7	
sd	5·3		5·0		5·6		4·9	
Country of birth, *n* (%)								
Australia	305	65·0	122	77·7	91	58·3	92	59·0
Other	164	35·0	35	22·3	65	41·7	64	41·0
Educational attainment, *n* (%)								
High school or less	161	34·3	74	47·1	48	30·8	39	25·0
TAFE certificate/diploma	177	37·7	65	41·4	58	37·2	54	34·6
University	131	27·9	18	11·5	50	32·0	63	40·4
Employment status, *n* (%)								
Employed	280	59·7	82	52·2	93	59·6	105	67·3
Unemployed	189	40·3	75	47·8	63	40·4	51	32·7
Marital status, *n* (%)								
Not married	46	9·8	24	15·3	15	9·6	7	4·5
Married/	423	90·2	133	84·7	141	90·4	149	95·5
Smoking status, *n* (%)								
Non-smoker	309	65·9	83	52·9	114	73·1	112	71·8
Current/past smoker	160	34·1	74	47·1	42	26·9	44	28·2
Prepregnancy BMI, kg/m^2^								
Mean	25·0		25·7		24·8		24·7	
sd	5·6		5·7		5·4		5·7	
DGI score								
Mean	47·5		36·7		48·7		57·2	
sd	9·5		6·5		2·3		3·2	
Child characteristic								
Child sex								
Boy	229	48·8	81	51·6	79	50·6	69	44·2
Girl	240	51·2	76	48·4	77	49·4	87	55·8
Infant feeding practices								
Breast-feeding duration (months)								
Median	6·1		3·8		7·4		7·1	
IQR	0·6–9·8		0·3–4·8		1–12·1		1·1–10·7	
Breast-feeding duration								
< 6 months	284	60·6	123	78·3	79	50·6	82	52·6
≥ 6 months	185	39·5	34	21·7	77	49·4	74	47·4
Timing of solids introduction								
< 6 months	233	49·7	86	54·8	81	51·9	66	42·3
≥ 6 months	236	50·3	71	45·2	75	48·1	90	57·7
Feeding mode at 12 months								
Formula/ mixed feeding	395	84·2	144	91·7	121	77·6	130	83·3
Breast-feeding	74	15·8	13	8·3	35	22·4	26	16·7

DGI score: 0 to 70, higher score indicates better diet quality. DGI tertiles scores: DGI tertile 1, 13·8–44·6; DGI tertile 2, 44·7–52·9; DGI tertile 3, 52·9–66·6. DGI, 2013 Dietary Guideline Index; TAFE, technical and further education.

### Maternal diet quality in pregnancy and associations with infant feeding practices

Table 2 presents the results of the linear and logistic regressions for associations between maternal DGI score and infant feeding practices. For DGI score, the crude analysis revealed that DGI score was positively associated with longer breast-feeding duration (months: *β* 0·14, 95 % CI 0·08, 0·20; < 6 *v*. ≥ 6 months: OR 1·06, 95 % CI 1·03, 1·08). For every one-unit increase in DGI score, women were more likely to continue breast-feeding for a further 0·14 months, which equates to approximately 4 days, or women had 6 % higher odds of breast-feeding for ≥ 6 months. Significant associations remained in the adjusted analyses; however, the effect sizes were slightly smaller. Significant positive associations were also observed for DGI score and feeding mode at age 12 months (OR 1·04, 95 % CI 1·01, 1·07). For every one-unit increase in DGI score, women had 4 % higher odds of breast-feeding their child at age 12 months than formula feeding or mixed feeding. No evidence of association was found for the DGI score and timing of solids introduction.

**Table 2.** Linear and logistic regression analyses investigating associations between maternal diet quality in pregnancy and infant feeding practices among women from the healthy beginnings trial (*n* 469) (Regression coefficient and 95 % CI)

DGI score	Breast-feeding duration (months)				Breast-feeding duration (≥ 6 *v*. < 6 months)				Solids introduction (≥ 6 *v*. < 6 months)				Feeding mode at 12 months (Breast-feeding *v*. formula/mixed)			
	*β*	95 % CI	*P*	*P* ^*^	OR	95 % CI	*P*	*P* ^*^	OR	95 % CI	*P*	*P* ^*^	OR	95 % CI	*P*	*P* ^*^
Overall																
Crude	0·14	0·08, 0·20	< 0·001		1·06	1·03, 1·08	< 0·001		1·01	0·99, 1·03	0·15		1·04	1·01, 1·07	0·01	
Adjusted	0·09	0·03, 0·15	0·006		1·04	1·02, 1·07	0·001		1·01	0·99, 1·03	0·45		1·03	1·00, 1·07	0·03	
DGI by tertile																
Crude				< 0·001				< 0·001								0·04
1 (Ref·)																
2	3·54	2·13, 4·96	< 0·001		3·53	2·15, 5·78	< 0·001						3·20	1·62, 6·34	0·001	
3	3·26	1·84, 4·67	< 0·001		3·30	2·01, 5·41	< 0·001						2·24	1·11, 4·56	0·03	
Adjusted				0·01				0·01								0·1
1 (Ref·)																
2	2·62	1·19, 4·04	< 0·001		2·80	1·66, 4·73	< 0·001						3·17	1·55, 6·51	0·002	
3	1·95	0·48, 3·41	0·01		2·26	1·33, 3·86	0·003						2·06	0·97, 4·39	0·06	

Crude models adjusted for intervention allocation. Adjusted models adjusted for maternal age, country of birth, educational attainment, employment status, marital status, smoking status, child sex, prepregnancy BMI and intervention allocation. *β*, regression coefficient; DGI tertile scores, median (IQR): DGI tertile 1, 37·4 (33·8–41·9); DGI tertile 2, 48·6 (46·7–50·4); DGI tertile 3, 56·9 (54·5–59·2). *Indicates *P*-trend. Significant at *P* < 0·05. DGI, 2013 Dietary Guideline Index.

When DGI score was analysed as tertiles, the crude analysis found that relative to women in DGI tertile 1, women in DGI tertiles 2 or 3 had 3·54 (95 % CI 2·13, 4·96) or 3·26 (95 % CI 1·84, 4·67) higher odds of having a longer duration of breast-feeding in months, respectively (Table 2). After adjusting for covariates, significant positive associations remained but with diminished effect sizes. Trend analysis showed a positive linear dose–response relationship between DGI tertiles and breast-feeding duration (crude: *P* < 0·001; adjusted: *P* = 0·01). Similar findings were found when breast-feeding duration was analysed as a binary variable (< 6 months v. ≥ 6 months). For feeding mode at age 12 months, compared with DGI tertile 1, women in DGI tertiles 2 or 3 were 3·20 (95 % CI 1·62, 6·34) or 2·24 (95 % CI 1·11, 4·56) times more likely to breastfeed their child for 12 months than to formula feed or mixed feed. Associations and effect sizes remained similar in the adjusted analysis. Trend analysis found a positive linear dose–response relationship between DGI tertiles and feeding mode in the crude model (*P* = 0·04) but not in the adjusted model (*P* = 0·1).

### Stratified analyses

**Figure 1.**
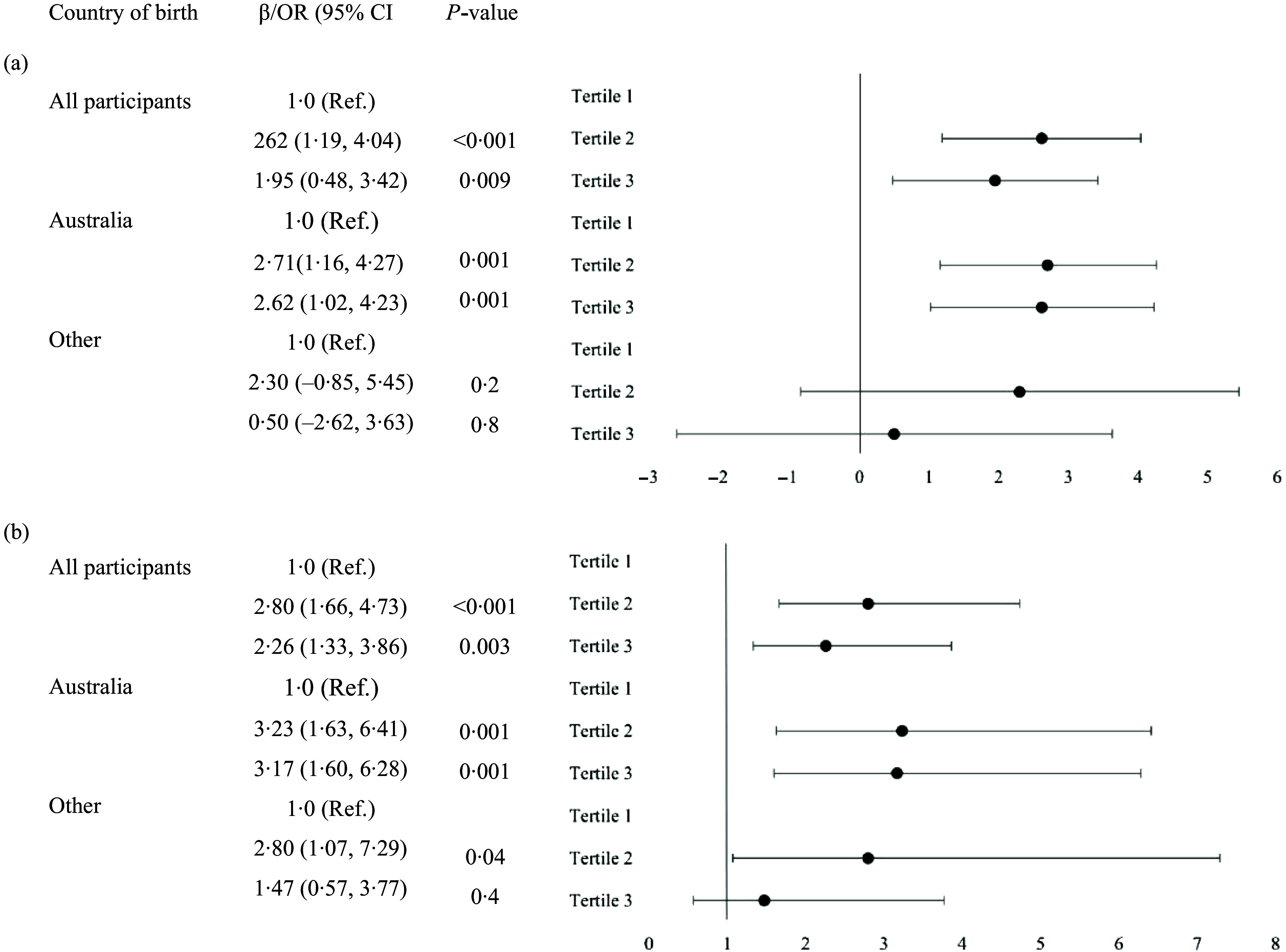
Forest plot of the associations between DGI tertile and breast-feeding duration in months (a) and ≥ 6 months *v*. < 6 months (b) stratified by country of birth with adjustment for covariates.

Stratified analyses showed the associations between DGI score (continuous score or tertiles) and breast-feeding duration (in months or < 6 *v*. ≥ 6 months) were significant among women born in Australia but not in women born overseas (DGI continuous score: breast-feeding in months: all participants—*β* 0·09, 95 % CI 0·03, 0·15; Australia—*β* 0·13, 95 % CI 0·06, 0·19; Other—*β* –0·03, 95 % CI –0·17, 0·11; < 6 *v*. ≥ 6 months: all participants—OR 1·04, 95 % CI 1·02, 1·07; Australia—OR 1·06, 95 % CI 1·03, 1·10; Other—OR 1·0, 95 % CI 0·96, 1·04; see Figure 1 for results for DGI tertiles). The likelihood ratio test revealed significant differences in stratum-specific effect sizes between women born in Australia and overseas (*P* < 0·05). This suggests that the significant positive associations between DGI score and longer breast-feeding duration were moderated or differed by maternal country of birth.

### Sensitivity analyses

Imputing the missing covariates (*n* 1 to *n* 29 missing, 6·8 % overall missingness) increased the effect sizes slightly for the association between DGI score and most infant feeding variables, and associations were in the same direction (online Supplementary Table 5).

## Discussion

This is the first study to investigate associations between maternal diet quality in pregnancy and infant feeding practices in Australian women. We found that a better maternal diet quality in pregnancy was associated with a longer breast-feeding duration. However, this association was observed in Australian-born women and not overseas-born women. We also found that a better diet quality during pregnancy was associated with a higher odds of breast-feeding than formula or mixed feeding when infants were 12 months old, but it was not associated with the timing of solids introduction. When maternal diet quality was categorised into tertiles, we found a positive linear dose–response relationship between maternal diet quality and breast-feeding duration.

We found that a better maternal diet quality in pregnancy was associated with longer breast-feeding duration, which is consistent with the few studies that have assessed postpartum maternal diet quality^(16,17)^. For example, a study reported that 751 US women with a high Food Source Quality score were 24 % less likely to stop breast-feeding before 6 months than women with a moderate Food Source Quality score^(16)^. Notably, that study used a similarly brief FFQ to collect maternal dietary data, adding weight to the comparability of our results. Another study of 1797 Finnish families found that women with a high Index of Diet Quality score at 4 months postpartum breastfed for longer than women with a low score^(17)^. Despite our findings showing a positive linear dose–response relationship between maternal diet quality in pregnancy and breast-feeding duration in the *P*-trend analysis, it is worth noting that the effect sizes for DGI tertile 3 were smaller than those of tertile 2. This is attributable to the shorter breast-feeding duration found in the highest DGI tertile compared with the mid DGI tertile. Further research is required to understand the dose–response relationship between maternal diet quality in pregnancy and breast-feeding duration. We also found that a better maternal diet quality in pregnancy was associated with higher odds of breast-feeding than formula or mixed feeding when infants were 12 months old. This finding aligns with a study of 360 women from the USA that found that women who consumed ≥ 3 servings of fruits and vegetables daily were more likely to breastfeed for 1 year compared with women who consumed fewer servings of fruits and vegetables^(34)^. Likewise, another study of 149 US women reported that women with lower fat and higher fruit intakes were more likely to breastfeed to 6 months postpartum^(35)^.

Several hypotheses may explain the beneficial role of a better maternal diet quality during pregnancy in promoting breast-feeding. It is possible that women who follow national dietary recommendations are more likely to follow recommendations for breast-feeding duration. Another potential reason is that women with a better diet quality during pregnancy may have better mental health or less stress^(36)^, which has been shown to be linked with a longer breast-feeding duration^(37)^. Alternatively, women with a healthier diet during pregnancy may be less likely to consume processed foods exposed to endocrine-disrupting chemicals, such as per- and polyfluoroalkyl substances and bisphenols, through plastic food packaging^(38)^. These chemicals have been found in breast milk, and prior research has shown that exposure to these chemicals could reduce breast-feeding duration through endocrine system disruption (e.g. impaired mammary gland development, lactogenesis and endocrine signalling)^(39)^.

To the authors’ knowledge, this is the first study to report that the association between better maternal diet quality and longer breast-feeding duration is moderated by maternal country of birth, with significant associations found in women born in Australia but not overseas. Our finding reflects the ethnic and cultural differences in maternal diet quality or breast-feeding duration that have been extensively reported^(40,41)^. Our finding is consistent with an Australian^(18)^ study and a Portuguese^(42)^ study that identified that women born overseas were more likely to breastfeed for longer compared with women born in Australia and Portugal, respectively. The longitudinal Portuguese study also reported that migrant women breastfed for longer regardless of the time they had resided in Portugal^(42)^, which emphasises the importance of ethnic and cultural differences.

Other maternal socio-demographic factors could support our finding concerning maternal diet quality in pregnancy and breast-feeding duration. Research has shown that women who are older and tertiary educated have a better diet quality than women who are younger and with no tertiary education^(40,43)^. In our study, women with a high diet quality were more likely to be older and tertiary educated. Previous research has shown that women who are older are more likely to be tertiary educated, have greater levels of nutrition knowledge^(44)^ and could, therefore, have greater awareness or knowledge of optimal infant feeding practices.

Maternal diet quality in pregnancy was not associated with the timing of solids introduction. Most prior research has used one variable to investigate the timing of solids introduction^(7)^. Few studies have explored maternal diet quality and timing of solids introduction. However, a Finnish study involving 1797 women, which assessed the timing of solids introduction as a continuous variable, found that women with a better postpartum diet quality introduced solids almost 1 month later than women with a poor diet quality^(17)^, which contrasts with the present study’s finding. The lack of a significant association in our study could be because we used a categorical (*v*. a continuous) variable to assess the timing of solids introduction or because we assessed maternal diet quality at a different time point (i.e. pregnancy *v*. postpartum).

Our study has several strengths. Our study is the first to explore the relationship between maternal diet quality in pregnancy and infant feeding practices. We also found the relationship between maternal diet quality in pregnancy and breast-feeding duration was moderated by maternal country of birth. In contrast to previous research involving the over-representation of highly educated women, we used data from the Healthy Beginnings Trial, which comprises women from diverse educational and cultural backgrounds, supporting the generalisability of our study’s findings to the wider Australian population. We also have some limitations to acknowledge. The dietary assessment tool used to assess maternal diet was relatively brief. Consequently, the full DGI score could not be calculated. Modifications to diet quality indices are common because of the different dietary assessment tools used^(45)^. However, we captured most key dietary intakes representative of encouraged and discouraged foods and relevant to health outcomes such fruits and vegetables and discretionary foods. We were also able to detect significant associations between maternal DGI score and breast-feeding, supporting the utility of using a short FFQ to derive a DGI score, similar to other studies^(46)^. Future research could replicate this analysis by using repeated measures of 24-hour recall data. In addition, only some of the dietary questions in the dietary assessment tool had been previously validated^(26)^, which could have implications for its reliability. The DGI was modified based on the availability of dietary data to enable the calculation of the diet quality scores. These omissions could make comparison of the results with other diet quality research challenging^(47)^. Parental reporting was used to determine the infant feeding variables, so parental reporting bias cannot be dismissed. However, infant feeding data were collected at several time points to help minimise this bias. As data on infant feeding practices were reported retrospectively by parents, there is a potential for recall bias. The current study did not assess exclusive breast-feeding; it would be desirable for future research to examine how maternal diet quality influences exclusive breast-feeding. Finally, there were differences in the excluded and included samples, with the excluded sample having a higher proportion of women who were younger, unmarried and unemployed. Inclusion of the excluded sample may have possibly weakened observed associations given that evidence has shown that women with these characteristics are less likely to practice infant feeding in line with recommendations^(48)^.

The findings from this study provide new evidence to support the initiation of dietary interventions in pregnancy, and such interventions should be tailored to cultural and ethnic groups. Nevertheless, additional research is needed to confirm our findings in wider population groups. Given adverse diet quality is also evident in preconception^(49)^, further studies could explore the relationship between maternal diet quality in preconception and infant feeding practices. Our understanding of mechanisms linking maternal diet quality and breast-feeding duration is limited; future research could explore the potential mechanisms.

In summary, in an Australian cohort, a better maternal diet quality in pregnancy was associated with longer breast-feeding duration in Australian women and not women born overseas. In addition, a better maternal diet quality was associated with a higher likelihood of breast-feeding *v*. formula or mixed feeding. Maternal diet quality was not associated with the timing of solids introduction. Our results add to the evidence supporting the implementation of dietary interventions to improve maternal diet quality in pregnancy, particularly in Australian-born women. Such interventions could potentially result in beneficial infant feeding outcomes. Moreover, interventions aimed at improving infant feeding practices should be initiated from pregnancy and target women born in Australia with poor diet quality.

## Supporting information

Sexton-Dhamu et al. supplementary material Sexton-Dhamu et al. supplementary material

## Supplementary material

For supplementary material/s referred to in this article, please visit https://doi.org/10.1017/S000711452500025X
